# Targeting STAT3 in Cancer Immunotherapy

**DOI:** 10.1186/s12943-020-01258-7

**Published:** 2020-09-24

**Authors:** Sailan Zou, Qiyu Tong, Bowen Liu, Wei Huang, Yan Tian, Xianghui Fu

**Affiliations:** 1 Division of Endocrinology and Metabolism, National Clinical Research Center for Geriatrics, State Key Laboratory of Biotherapy and Cancer Center, West China Hospital, Sichuan University and Collaborative Innovation Center of Biotherapy, Chengdu, 610041 Sichuan China; 2 College of Life Sciences, Sichuan University, Chengdu, 610041 Sichuan China; 3 Department of Integrated Traditional Chinese and Western Medicine, Sichuan Provincial Pancreatitis Centre and West China-Liverpool Biomedical Research Centre, West China Hospital, Sichuan University, Chengdu, 610041 China

**Keywords:** STAT3, Cancer, Immunosuppression, Immunotherapy, Immune checkpoint blockade, CAR-T

## Abstract

As a point of convergence for numerous oncogenic signaling pathways, signal transducer and activator of transcription 3 (STAT3) is central in regulating the anti-tumor immune response. STAT3 is broadly hyperactivated both in cancer and non-cancerous cells within the tumor ecosystem and plays important roles in inhibiting the expression of crucial immune activation regulators and promoting the production of immunosuppressive factors. Therefore, targeting the STAT3 signaling pathway has emerged as a promising therapeutic strategy for numerous cancers. In this review, we outline the importance of STAT3 signaling pathway in tumorigenesis and its immune regulation, and highlight the current status for the development of STAT3-targeting therapeutic approaches. We also summarize and discuss recent advances in STAT3-based combination immunotherapy in detail. These endeavors provide new insights into the translational application of STAT3 in cancer and may contribute to the promotion of more effective treatments toward malignancies.

## Introduction

Dysregulation of immune checkpoints is a protective mechanism used by a number of malignancies to escape from the immune surveillance allowing for cancer development [1]. This has inspired the idea of boosting the host immune response as an anti-cancer therapy. Indeed, the blockage of immune checkpoints, including programmed cell death protein 1 (PD-1), programmed cell death 1 ligand 1 (PD-L1) and cytotoxic T-lymphocyte-associated protein 4 (CTLA-4), improves clinical outcomes in subsets of patients with cancers previously considered to be essentially untreatable [2–4]. In order to expand the array of treatable cancers as well as increase the number of patients that respond to the therapy, novel therapeutic targets and new molecules/strategies should be urgently identified and developed for immunotherapy appropriate for the clinical use.

The signal transducer and activator of transcription (STAT) proteins are a family of cytoplasmic transcription factors which share an overall general structure, organized into functional modular domains. The mammalian STAT family comprises STAT1, STAT2, STAT3, STAT4, STAT5a, STAT5b and STAT6 that mediate multiple intracellular signaling pathways [5]. Among them, STAT3 is involved in numerous biological processes including cell proliferation, survival, differentiation, and angiogenesis [6, 7]. In normal cells, transient activation of STAT3 (predominantly by phosphorylation) transmits transcriptional signals from cytokines and growth factor receptors at the plasma membrane to the nucleus [5]. In contrast, STAT3 becomes hyperactivated in the majority of human cancers and is generally associated with poor clinical prognosis [8]. Therefore, it is not surprising that STAT3 signaling pathway has long been recognized as a potential therapeutic target for cancer therapy owing to their roles in tumor formation, metastasis and drug resistance [9–12]. Moreover, accumulating evidence reveals that STAT3 hyperactivation can mediate tumor-induced immunosuppression at many levels [13, 14]. Given the similarities between tumorigenesis and STAT3-dependent immunity, new therapeutic strategies that target STAT3 signaling pathway may open up new avenues for long-lasting and multilayered tumor control.

This review outlines the role of the STAT3 pathway in tumor immunity, summarizes the recent progress in STAT3-centered anti-cancer approaches, and highlights future directions for the clinical immunotherapy.

## The STAT3 signaling pathway

**Fig. 1:**
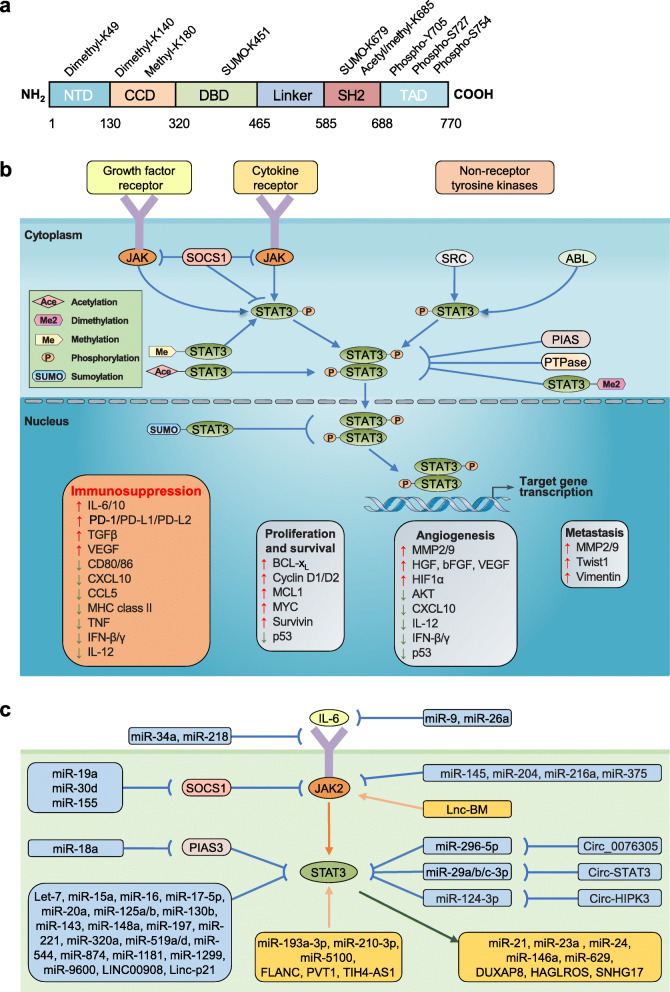
The domain structure and signaling pathway of STAT3. **a** Schematic domain structure of STAT3. STAT3 is characterized by the presence of six different functional domains, including an amino-terminal domain (NTD) for cooperative binding of STAT proteins to multiple consensus DNA sites, a coiled-coil domain (CCD) for recruitment of STAT3 to the receptor as well as subsequent phosphorylation, dimerization and nuclear translocation, a DNA-binding domain (DBD) for recognizing and binding to a specific consensus DNA sequence, a linker domain for connecting the DBD with the SRC homology 2 (SH2) domain, a SH2 domain for recruitment and activation as well as dimerization of the STAT3 molecule by interacting with phosphorylated tyrosine residues in the opposing subunit, and a carboxyl-terminal transactivation domain (TAD). **b** STAT3 signaling pathway. STAT3 is activated by upstream growth factor kinases and cytokine receptors. Non-receptor tyrosine kinases such as SRC and ABL can also lead to constitutive activation of STAT3. Phosphorylated STAT3 dimerizes and translocates to nucleus, which causes the transcription of target genes including immunosuppression, angiogenesis, metastasis, proliferation and survival. The signaling pathway can be inhibited by SOCS proteins, PIAS proteins, and protein tyrosine phosphatases (PTPases), etc. **c** Interplay between noncoding RNAs and STAT3 signaling pathway. On the one hand, miRNAs and lncRNAs can regulate STAT3 activation through not only directly targeting STAT3, but also targeting the components of the STAT3 signaling pathway, such as IL-6, JAK2, SOCS1 and PIAS3; CircRNAs usually regulate STAT3 by acting as sponges for miRNAs. On the other hand, STAT3 is able to regulate miRNAs and lncRNAs expression in many ways.

STAT3 is a protein consisting of 770 amino acids and characterized by the presence of 6 functionally conserved domains, including the amino-terminal domain (NH2), the coiled-coil domain (CCD), the DNA-binding domain (DBD), the linker domain, the SRC homology 2 (SH2) domain, and the carboxyl-terminal transactivation domain (TAD) (Fig. 1a). Among them, SH2 is the most highly conserved STAT domain and plays a crucial role in signaling via binding to specific phosphotyrosine motifs [15]. In an unstimulated cell, STAT3 is tightly regulated by negative modulators to maintain an inactive state in the cytoplasm. These modulators include members of the protein inhibitor of activated STAT (PIAS), suppressor of cytokine signaling (SOCS) families, protein tyrosine phosphatases (SHP1, SHP2, PTPN1, PTPN2 PTPRD, PTPRT and DUSP22), and ubiquitin enzymes [8]. In response to stimuli, STAT3 becomes activated mainly by direct phosphorylation at tyrosine (705) and serine (727) residues induced by its upstream ligands including Janus kinases (JAKs), tyrosine kinases, cytokines and several non-receptor tyrosine kinases such as SRC and ABL; the phosphorylation induces dimerization of STAT3 proteins followed by nuclear translocation, DNA binding, and eventually execution of their nuclear functions [15].

Beyond phosphorylation, other posttranslational modifications (i.e. acetylation, methylation, and sumoylation) can also regulate STAT3 transcriptional activity through altering STAT3 phosphorylation, and thus add another layer of complexity for STAT3 hyperphosphorylation in cancers (Fig. 1b). For instance, acetylation at several lysine residues within both the NH2 and SH2 domains, primarily mediated by the CBP/p300 acetyltransferase, can enhance STAT3 transactivating potential, which is associated with increased dimer stabilization, tyrosine 705 phosphorylation, nuclear translocation, and localized histone hyperacetylation of target promoters [16]. In contrast, deacetylation by several deacetylases, such as HADC1-3, SIRT1 and Loxl3, inhibits transcription of STAT3 targets [17–19]. The dynamic balance of acetylation and deacetylation plays a role in STAT3 activation and is involved in various cellular events. Similarly, methylation and sumoylation are also emerging as important regulatory mechanisms for STAT3 activity. SMYD2-dependent methylation of STAT3 contributes to the hyperphosphorylation of STAT3, whereas EZH2- and SET9-dependent dimethylation of STAT3 inhibits the activity of DNA-bound STAT3 dimers [20–22]. Sumoylation at the lysine 451 of STAT3 by SUMO2/3 can promote its interaction to the nuclear phosphatase TC45, thereby restraining phosphorylated STAT3 in the nucleus, while de-sumoylation by SENP3 leads to the hyperphosphorylation of STAT3 [23]. However, androgen receptor degradation enhancer ASC-J9 can inhibit the STAT3 phosphorylation via inducing the sumoylation of STAT3 at lysine 679 [24].

Additionally, increasing evidence suggests that noncoding RNAs (ncRNAs) can directly or indirectly modulate STAT3 activity (Fig. 1c). As the most extensively studied ncRNAs, numerous microRNAs (miRNAs) have been shown to target STAT3 directly and certain components of STAT3 signaling pathway (IL-6, JAK2, SOCS1, PIAS3, etc.), thereby modulating STAT3 expression and activation [8, 25–29]. For instance, miR-125b-5p can directly target STAT3 and inhibit its expression [27], while miR-218 indirectly suppresses STAT3 activation by targeting IL-6 receptor and JAK3 [28]. More recently, it has shown that exosome-mediated transfer of certain miRNAs, such as miR-193a-3p, miR-210-3p and miR-5100, can promote metastasis of lung cancer by enhancing STAT3 activity [29], although the molecular mechanisms await further investigation. Likewise, long non-coding RNAs (lncRNAs) can modulate the expression and activation of STAT3 directly or indirectly through multiple mechanisms [30–33]. For instance, lincRNA-p21 can inhibit the STAT3 transcriptional activity via directly binding to STAT3 [30]. Lnc-BM can bind to the JH2 domain of JAK2, which increases JAK2 activation, and thus indirectly enhances activity of STAT3 [32]. FLANC, a novel primate-specific lncRNA, has shown to upregulate and prolong the half-life of phosphorylated STAT3, but not total STAT3, albeit the underlying mechanism remains unknown [34]. Intriguingly, a recent study revealed that LINC00908-encoded polypeptide ASRPS can directly bind to the CCD domain of STAT3, and thus reduce STAT3 phosphorylation [35]. In general, circular RNAs (circRNAs) can modulate gene expression by acting as sponges of endogenous miRNAs. It has shown that circ-HIPK3, circ_0076305 and circ-STAT3 positively modulate STAT3 signaling by sponging miR-124-3p, miR-296-5p, and miR-29a/b/c-3p, respectively [36–38]. In parallel, STAT3 has a capacity of regulating ncRNAs directly or indirectly. The regulation of miR-21 by STAT3 has been extensively studied. On the one hand, STAT3 can directly regulate miR-21 transcription in myeloma cells by binding to its upstream enhancer region [39]. On the other hand, STAT3 can upregulate miR-21 through increasing IL-6 expression [40]. The regulatory effect of STAT3 in lncRNAs is also emerging. STAT3 can directly bind to the promoter region of certain lncRNAs, such as SNHG17, DUXAP8 and HAGLROS, and thus contributes to their overexpression in cancers [41–43].

As described above, STAT3 activity can be influenced by many factors such as numerous post-translational modifications and multiple ncRNAs regulation. These complexities, together with the fact that the STAT3 signaling pathway is responsive to a great variety of cellular stresses and stimuli [44], pose difficulties in our understanding of abnormal hyperactivated STAT3 in cancers. Future investigations delineating the regulatory network of STAT3 will likely facilitate the clinical translation of STAT3-based therapies for human malignancy.

## STAT3-driven tumor immunosuppression

The tumor microenvironment (TME) is a highly complex and heterogenous ecosystem consisting tumor-infiltrating immune cells, cancer-associated fibroblasts (CAFs), smooth muscle cells, endothelial cells, and the tumor cells [45, 46]. It is becoming increasingly evident that TME can promote the progression of cancer and mediate therapeutic resistance, particularly against cancer immunotherapy [47, 48]. Gathered evidence suggests that STAT3 becomes hyperactivated not only in cancer cells themselves but also in immune cells and CAFs within the TME [13, 49–52]. The hyperactivation of STAT3 in TME compartments might have a significant impact on anti-tumor immunity through various mechanisms (described below in more detail).

### STAT3 in tumor cells

In tumor cells *per se*, hyperactivated STAT3 decreases the expression of immune-stimulating factors including interferons (IFNs), pro-inflammatory cytokines (IL-12, TNF-α) and chemokines (CCL5, CXCL10), while increases the expression of certain cytokines and growth factors (IL-6, IL-10, TGFβ, and VEGF), thereby exerting profound immune effects (Fig. 1b) [53]. For instance, STAT3 can suppress the secretion of type 1 IFNs (IFN-Is) and IFN-I-responsive genes via multiple actions, such as attenuating the activation of IFN-I signaling, reducing the expression of ISGF3 components, and impairing the potential of ISGF3 transactivation [54, 55].

In tumor cells, STAT3 often interacts with other signaling pathways, such as NF-κB, to confer robustness for tumor progression [44, 56, 57]. NF-κB signaling is of importance for both inflammation-induced carcinogenesis and anti-tumor immunity [57]. NF-κB (especially RELA) can upregulate a spectrum of targets involved in chronic inflammation and cancer initiation such as cyclooxygenase 2, IL-6, IL-23, and IL-1β [44]. Several layers of STAT3-NF-κB crosstalk have been identified thus far: (1) both NF-κB and STAT3 are frequently activated in the same tumor cells and TME-associated infiltrating immune cells, and share a wide range of common targets that participate in cell proliferation, metastasis, anti-apoptosis, and angiogenesis [56]; (2) STAT3 can prolong nuclear retention of RELA through p300-mediated acetylation, leading to the persistent activation of NF-κB [58]; (3) many cytokines (i.e. IL-6) can in turn simultaneously activate STAT3 and NF-κB [57]; (4) it has recently been demonstrated that NF-κB activity in pancreatic CAFs shielded cancer cells from immune attack by increasing CXCL12 expression [59]. Given the well-known feedforward loop between CXCL12 and STAT3 [60, 61], it is possible that STAT3 contributes to NF-κB-mediated immune evasion by this vicious cycle.

### STAT3 in immune cells

STAT3 also plays a pivotal role in a plethora of tumor-infiltrating immune cells that predominantly comprise the TME and recent comprehensive reviews have covered this topic [62–64]. Here we would only like to briefly mention the diverse functions of STAT3 in immune cell milieu, together with some recent advances that throw new lights on our understanding of its extremely sophisticated regulation.

Hyperactivation of STAT3 in tumor-infiltrating immune cells causes immunosuppression by inhibiting both innate and adaptive immune responses. In brief, excessive STAT3 activity in innate immune cell subsets may impair the production of pro-inflammatory mediators such as IFNγ, dampen antigen presentation, and inhibit the tumor-killing activities of effector cells. In adaptive immune subsets, elevated STAT3 activity has the ability to inhibit the accumulation of effector T cells, thereby restraining their anti-tumor effects [62–64]. Interestingly, some recent studies suggest previously unknown functions of STAT3 in tumor immunity. For instance, placental growth factor (PlGF) [65] and Cxxc finger protein 1 (Cxxc1) [66] can act as key upstream regulators of STAT3 signaling, which subsequently contributes to the differentiation and function of Th17 cells. STAT3 mediates the major impact of β2 adrenergic receptor on the immunosuppressive potential of myeloid-derived suppressor cells (MDSCs) in the TME [67]. In glioblastoma-infiltrating tumor-associated macrophages, STAT3 acts as a positive regulator of aryl hydrocarbon receptor (AHR) and thus increases the recruitment of tumor-associated macrophages and tumor growth [68]. It has been shown that STAT3 modulates the abundance and function of regulatory T (Treg) cells in response to radiation therapy in head and neck cancer, suggesting that STAT3 inhibition may be beneficial for patients receiving radiation [69].

### STAT3 in CAFs

CAFs are the key component of the tumor stroma and contribute to cancer progression and treatment failure by modifying the extracellular matrix, secreting soluble factors, supporting angiogenesis and metastasis, and inhibiting anti-tumor immune responses [70]. There is a growing body of evidence to support that STAT3 can be activated in CAFs by numerous cytokines including leukemia inhibitory factor (LIF) [71]. This STAT3 hyperactivation enables CAFs to produce various immunosuppressive factors such as IL-6, TGFβ, EGF, VEGF, and CCL2, thereby contributing to the pro-oncogenic phenotype of these fibroblasts [72, 73]. Moreover, a recent study revealed that increased phosphorylation of STAT3 in CAFs is associated with reduced overall survival in colorectal cancer patients, and STAT3 activation in CAFs enhances intestinal tumor growth *in vivo* [74], exemplifying the importance of STAT3 activation in CAFs for cancer initiation and progression.

### STAT3-mediated crosstalk between cancer cells and diverse cell subsets in the TME

**Fig. 2:**
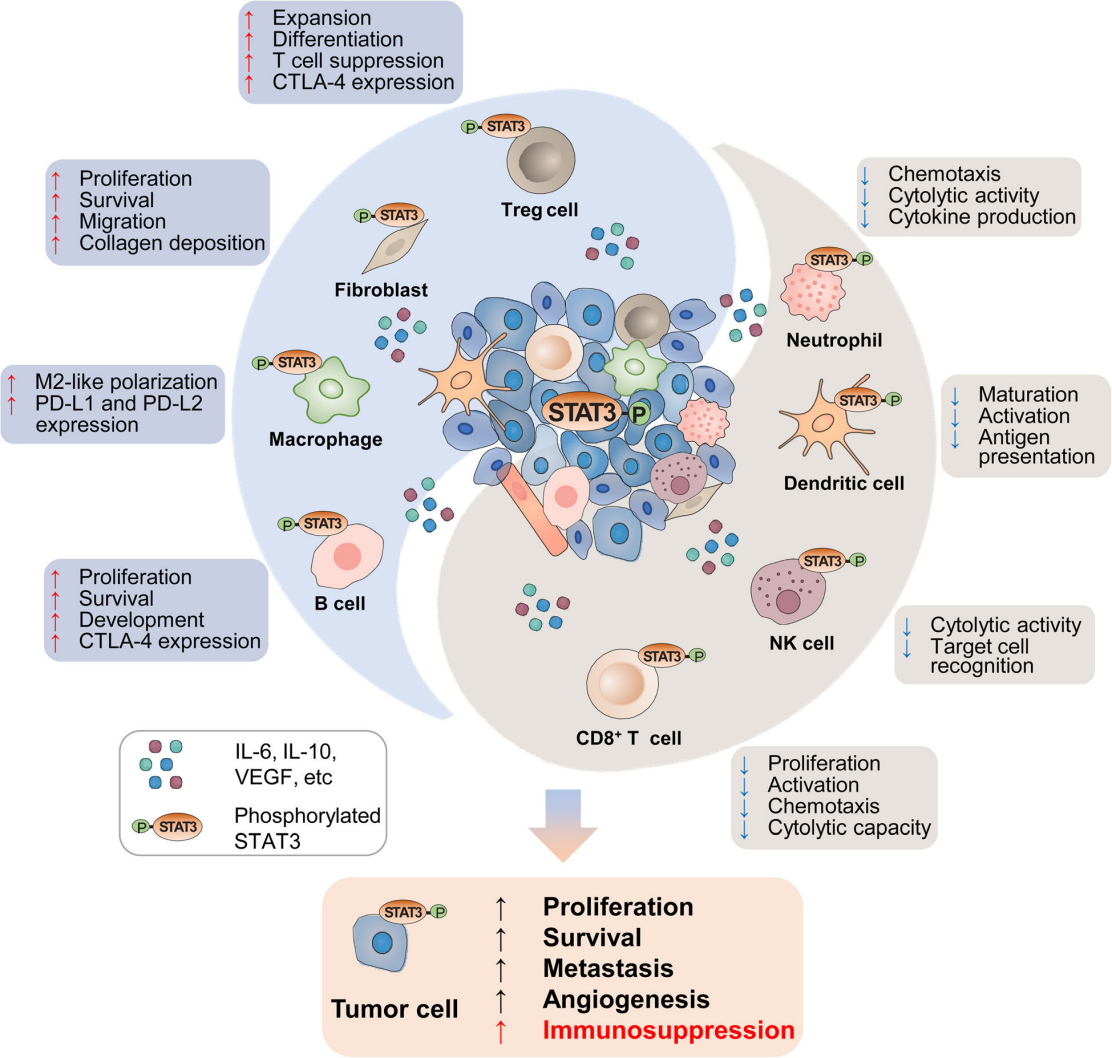
STAT3 induces the immunosuppression in the TME. STAT3 activity in tumor cells supports multiple hallmarks of cancer, including increased secretion of immunosuppressive factors such as IL-6, IL-10 and EGFR, which can activate STAT3 in the innate and adaptive immune cell subsets as well as CAFs in the TME. Likewise, immune cells and CAFs within the TME can release certain factors including IL-6, which subsequently enhance STAT3 signaling in tumor cells. Elevated STAT3 in the TME has dual effects. On the one hand, STAT3 favors the accumulation and enrichment of immunosuppressive Treg cells and B cells, as well as the polarization of M2-like macrophages, which instigate immune evasion. Particularly, STAT3 is a major driver for increased expression of immune checkpoint molecules (such as PD-L1, PD-L2 and CTLA-4) in these cells. On the other hand, STAT3 in CD8^+^ T cells, NK cells and neutrophils evokes restrained anti-tumor cytolytic activities. STAT3 can also inhibit the anti-tumor ability of DCs through dampening their maturation, activation and antigen presentation. Besides, STAT3 in CAFs can promote their proliferation, survival and migration, and drive the remodeling of tumor stroma for tumor progression. Collectively, STAT3 induces the immunosuppression in the TME, thereby promoting tumor progression with diminishing the anti-tumor immunity.

Aberrantly activated STAT3 can lead to tumor-induced immunosuppression via propagating the crosstalk between cancer cells and their immunological microenvironment. In tumor cells, hyperactivated STAT3 promotes the expression of immunosuppressive factors such as VEGF, IL-6, and IL-10 [53]. Meanwhile, these tumor-derived factors that also happen to be STAT3 activators could be transited to the TME, and thus enhance STAT3 signaling in various immune cell subsets and CAFs (Fig. 2).

In particular, STAT3 hyperactivation in tumor cells has a vital role in dendritic cells (DCs) maturation. DCs essentially are monocytes at a differentiated stage and the key antigen presenting cells of the immune system. As immune sentinels, DCs play an important role in the initiation of T-cell response against tumors, while immature DCs generally induce immune tolerance [75]. Hyperactivated STAT3 in tumor cells can suppress the expression of IL-12 and TNF-α, leading to a decrease in Bcl-2 expression in DCs [53]. STAT3 also represses the expression of major histocompatibility complex (MHC) class II complexes and co-stimulatory signals (CD80 and CD86), which are essential to the antigen presenting function of DCs [13]. Meanwhile, STAT3 inhibits DC maturation and innate immunity through negatively regulating the expression of CXCL10 and CCL5 [53]. Furthermore, the immunosuppressive factors such as IL-6, IL-10, and VEGF induced by STAT3 can inhibit DC generation through reducing protein kinase C beta II (PKCβII) expression [76]. Given that immature DCs cannot activate antigen-specific CD8^+^ T cells, activated STAT3 signaling in tumor cells reduces the anti-tumorigenic effector functions of CD8^+^ T cells.

What’s more, certain factors released by CAFs can modulate STAT3 signaling in other cell types in the tumor milieu. TGFβ, an evolutionarily conserved regulator of tumorigenesis, is a crucial driver of the activity of CAFs. Accumulating evidence suggests that TGFβ-stimulated CAFs increase the secretion of IL-6 and IL-11, which trigger GP130/STAT3 signaling in cancer cells and thus promote cancer metastasis and progression [77–80]. STAT3 is also involved in the crosstalk between CAFs and immune cells. For example, CCL2 secreted from CAFs with STAT3 hyperactivation can promote the recruitment of immunosuppressive MDSCs and hepatocarcinogenesis [72]. Moreover, the differentiation of these recruited MDSCs has been shown to be controlled in an IL-6/STAT3-dependent manner [81]. In addition, IL-6 derived from CAFs can activate STAT3 in DCs, which subsequently induce liver cancer immune escape through impairing T-cell proliferation and promoting Treg cells expansion [82]. STAT3 signaling in CAFs and other cells orchestrates stromal remodeling of the TME characterized by collagen fibrogenesis, collagen disorganization and fibroblast contractility; the remodeling of the TME is not only important for cancer cell migration and invasion, but also plays a critical role in resistance to therapeutic intervention [83, 84].

Overall, the outcome of STAT3-mediated crosstalk between cancer cells and tumor-infiltrating cells within the TME is to promote tumor growth and development, along with diminished anti-tumor immunity (Fig. 2).

## Targeting STAT3 for cancer immunotherapy

### Direct targeting of STAT3

Although targeting STAT3 has been extensively investigated for decades, this field still remains largely unexplored. The most common approach in targeting STAT3 directly is to prevent the formation of functional STAT3 dimers through disrupting the domains of SH2, DBD, or NTD [85, 86]. In general, direct inhibitors of STAT3 can be classified into three categories: peptides, small molecules and oligonucleotides. Studies of these inhibitors on pre-clinical cancer models are summarized in Table 1 [87–112] and relevant ongoing clinical trials are introduced in Table 2 [117–119].

**Table 1:** Studies of STAT3 inhibitors on pre-clinical cancer models

Therapy	Type	Agent	Cell line tested	Mouse model	Functional outcome	Ref
**Direct inhibitors**	Peptides	DBD-1	Melanoma, Myeloma	n/d	↑Apoptosis; ↓Proliferation	[87]
		ISS-610 prodrugs	BC	n/d	↑Apoptosis	[88]
		PY*LKTK	NIH3T3/v-Src or v-Ras	n/d	↓Transformation	[89]
	Small molecules	6o	BC, PC, PCa, NSCLC	n/d	↑Apoptosis; ↓Proliferation	[90]
		FLLL32	BC, PC	Xenograft: MDA-MB-231, PANC-1	↑Apoptosis; ↓Proliferation, Vascularization	[91]
		HJC0152	Glioblastoma	Xenograft: U87	↑Apoptosis; ↓Metastasis, Proliferation	[92]
		LL1	CRC	Xenograft: HCT116	↑Apoptosis; ↓Metastasis, Proliferation	[93]
		LLL-3	BC, Glioblastoma	Xenograft: U87	↑Apoptosis; ↓Metastasis, Proliferation	[94]
		LLL12	HCC	Xenograft: SNU398	↑Apoptosis; ↓Proliferation	[95]
		LYW-6	CRC	AOM/DSS induced CRC model; Xenograft: HCT116	↑Apoptosis; ↓Metastasis, Proliferation	[96]
		Nitidine chloride	Oral cancer	Xenograft: HSC3	↑Apoptosis; ↓Proliferation	[97]
		SD-36	BC, CRC, Leukemia, Lymphoma	Xenograft: MOLM-16, SUP-M2, SU-DHL-1	↑Apoptosis; ↓Proliferation	[98]
		Stattic	BC	n/d	↑Apoptosis	[99]
		STX-0119	n/d	Humanized NOG-dKO model	↑Anti-tumor immunity; ↓Proliferation	[100]
		S3I-1757	Melanoma	Xenograft: B16-F10	↓Proliferation	[101]
		S3I-201	BC	Xenograft: MDA-MB-231	↑Apoptosis; ↓Proliferation	[102]
		CPA-7	BC, CRC, Melanoma, PCa, NSCLC	Xenograft: CT26	↑Apoptosis; ↓Proliferation	[103]
		C48	BC, CML, Melanoma, PCa	Xenograft: MDA-MB-468, C3L5	↓Proliferation	[104]
		GPA512	PCa	Xenograft: DU145	↓Proliferation	[105]
		MMPP	BC, CRC, PCa, HCC, Lung, Ovary and Skin cancer	Xenograft: Patient-derived NSCLC, NCI-H460	↑Apoptosis; ↓Proliferation	[106]
	Oligonucleotides	InS3-54A18	BC, NSCLC	Xenograft: A549	↓Metastasis, Proliferation	[107]
		STAT3 hpdODN	CRC	n/d	↓Proliferation	[108]
**Indirect inhibitors**	JAK2	INCB16562	Leukemia	MPLW515L model	↓Proliferation	[109]
		TG101209	Leukemia	AML1-ETO9a leukemia model	↑Apoptosis; ↓Proliferation	[110]
	EGFR	JND3229	BaF3	Xenograft: BaF3-EGFR	↓Proliferation	[111]
	FGFR, VEGFR	ODM-203	Bladder cancer, NSCLC, GC	Xenograft: H1581, KMS11, RT4, SNU16	↑Anti-tumor immunity; ↓Metastasis, Proliferation	[112]
**Combination**	Direct inhibitor	HJC0152	BC, THP1	Xenograft: 4T1	↑Anti-tumor immunity; ↓Proliferation	[113]
	STING agonist	c-diAM (PS)2				
	JAK1/2 inhibitor	Ruxolitinib	PC	Xenograft: PANC02-H7	↑Anti-tumor immunity; ↓Proliferation	[114]
	Anti-PD-1 antibody	RMP1-14				
	SRC, ABL inhibitor	Dasatinib	n/d	*Tgfbr1/Pten* 2cKO model	↑Anti-tumor immunity; ↓Proliferation	[115]
	Anti-CTLA-4 antibody	9D9				
	VEGFR2 antibody	DC101	n/d	Xenograft: LLC, CT26	↓Proliferation; ↑Anti-tumor immunity, Vascular normalization	[116]
	STING agonist	cGAMP, RR-CDA				

*AOM/DSS* Azoxymethane/dextran sodium sulfate, *BC* Breast cancer, *CML* Chronic myelogenous leukemia, *CRC* Colorectal cancer, *GC* Gastric cancer, *HCC* Hepatocellular carcinoma, *LLC* Lewis lung carcinoma, *MPLW515L* Somatic mutations at codon 515 of the thrombopoietin receptor, *NSCLC* Non-small cell lung cancer, *PC* Pancreatic cancer, *PCa* Prostate cancer, *n/d* Not determined, h*pdODN* hairpin decoy oligodeoxynucleotide

**Table 2:** STAT3 inhibitors in currently on-going clinical trials

Therapy	Type	Agent	Indication	Phase	NCT number	Ref
**Direct inhibitors**	Small molecules	BBI608 (FDA approved)	Advanced malignancies	I/II	NCT01775423	NA
			CRC	III	NCT01830621	[117]
		Celecoxib* (FDA approved)	CRC	III	NCT00087256	NA
		C188-9	BC, CRC, HNSCC, HCC, NSCLC, GAC, Melanoma, Advanced cancer	I	NCT03195699	NA
		OPB-111077	Acute myeloid leukemia	I	NCT03197714	NA
			Advanced HCC	I	NCT01942083	NA
		OPB-31121	Advanced cancer, Solid tumors	I	NCT00955812	NA
			HCC	I/II	NCT01406574	NA
		OPB-51602	Malignant solid tumors	I	NCT01184807	NA
			Hematological malignancies	I	NCT01344876	NA
			Nasopharyngeal carcinoma	I	NCT02058017	NA
		Pyrimethamine* (FDA approved)	CLL, Small lymphocytic lymphoma	I/II	NCT01066663	NA
	Oligonucleotides	AZD9150	Lymphoma	I/II	NCT01563302	[118]
		STAT3 decoy	Head and neck cancer	0	NCT00696176	[119]
**Indirect inhibitors**	JAK1/2	AZD-1480	Solid tumors	I	NCT01112397	NA
		CYT 387	Myelofibrosis	I/II	NCT01423058	[120]
			PMF, Post-PV, Post-ET MF	III	NCT02101268	NA
		Ruxolitinib (FDA approved)	Myelofibrosis	II	NCT03427866	NA
	JAK2	LY2784544	Myeloproliferative neoplasms	II	NCT01594723	[121]
		SB1518	Myelofibrosis	III	NCT02055781	[122]
	EGFR	Cetuximab (FDA approved)	Metastatic CRC	I/II	NCT02117466	NA
		Panitumumab (FDA approved)	Advanced CRC	II	NCT03311750	NA
			Metastatic CRC	IV	NCT02301962	NA
	FGFR	Ponatinib (FDA approved)	CML	II	NCT04043676	NA
			CML, ALL	II	NCT04233346	NA
	IL-6R	Siltuximab (FDA approved)	Multiple myeloma	II	NCT03315026	NA
		Tocilizumab (FDA approved)	HCC	I/II	NCT02997956	NA
	VEGF	Bevacizumab (FDA approved)	Metastatic CRC	II	NCT02226289	NA
	VEGFR	Apatinib	Lung carcinoma	II	NCT03709953	NA
	VEGFR, PDGFR	Sorafenib (FDA approved)	Advanced HCC	IV	NCT02733809	NA
	VEGFR, PDGFR, c-KIT	Sunitinib (FDA approved)	Clear cell renal carcinoma	II	NCT03066427	NA
			Pancreatic neuroendocrine tumor metastatic	II	NCT02713763	NA
	SRC, ABL	Dasatinib (FDA approved)	Chronic-phase CML	IV	NCT01660906	[123]
	SRC	Bosutinib (FDA approved)	CML	II	NCT02810990	NA
		KX2-391	Bone-metastatic, Castration-resistant PCa	II	NCT01074138	[124]
**Combination**	Direct inhibitors and ICB	AZD9150, Durvalumab (anti-PD-L1)	NSCLC	II	NCT03334617	NA
			PC, CRC, NSCLC	II	NCT02983578	NA
			Advanced solid tumors, Metastatic HNSCC	I/II	NCT02499328	NA
			Diffuse large B-cell lymphoma	I	NCT02549651	NA
		BBI608, Nivolumab (anti-PD-L1)	Metastatic CRC	II	NCT03647839	NA
		BBI608, Pembrolizumab (anti-PD-1)	Metastatic CRC	I/II	NCT02851004	NA
	Indirect inhibitors and ICB	Apatinib, SHR-1210 (anti-PD-1)	Melanoma	II	NCT03955354	NA
		Bevacizumab, Atezolizumab (anti-PD-L1)	Unresectable HCC	III	NCT03434379	[125]
		Cetuximab, Pembrolizumab (anti-PD-1)	Recurrent or metastatic HNSCC	II	NCT03082534	NA
		Dasatinib, Ipilimumab (anti-CTLA-4)	GIST, Stage III /IV soft tissue sarcoma	I	NCT01643278	[126]
		Dasatinib, Nivolumab (anti-PD-L1)	Philadelphia chromosome positive ALL	I	NCT02819804	NA
		Ruxolitinib, Pembrolizumab (anti-PD-1)	Hematological malignancies	II	NCT04016116	NA
			Metastatic stage IV TNBC	I	NCT03012230	NA
		Sorafenib, BGB-A317(anti-PD-1)	HCC	III	NCT03412773	NA
		Sorafenib, Nivolumab (anti-PD-L1)	Advanced or metastatic HCC	II	NCT03439891	NA
	Indirect inhibitor and CAR-T	Tocilizumab, CAR-T 19	Lymphoblastic leukemia	NA	NCT02906371	NA

*ALL* Acute lymphoblastic leukemia, *BC* Breast cancer, Celecoxib* An FDA approved nonsteroidal anti-inflammatory drug, *CML* Chronic myelogenous leukemia, *CLL* Chronic lymphocytic leukemia, *CRC* Colorectal cancer, *HNSCC* Head and neck squamous cell carcinoma, *NA* Not available, *NSCLC* Non-small cell lung cancer, *HCC* Hepatocellular carcinoma, *GAC* Gastric adenocarcinoma, Pyrimethamine* An FDA approved anti-parasitic drug, *PMF* Primary myelofibrosis, *Post-PV* Post-polycythemia vera, *Post-ET MF* Post-essential thrombocythemia myelofibrosis, *PC* Pancreatic cancer, *PCa* Prostate cancer, *GIST* Gastrointestinal stromal tumor, *TNBC* Triple negative breast cancer

Peptides are usually designed based on the structure of amino acid residues in STAT3 protein and can be directed towards different domains. Phosphopeptide inhibitor (PY*LKTK), derived from the binding peptide sequence of the STAT3-SH2 domain, represents the first successful attempt to disrupt STAT3 dimerization [89]. However, the further development of peptide for the clinical use is currently limited due to their poor cellular permeability and lack of stability *in vivo*, and even the second-generation peptidomimetics are largely suffering from similar limitations [127].

Non-peptide small molecules capable of disrupting phosphorylation of STAT3 or STAT3-STAT3 dimerization have recently emerged as an attractive alternative approach to the above. These small molecule inhibitors usually selectively bind to the SH2, the DBD, or the NTD domain of STAT3 to block transcription of target genes [85]. BBI608 (Napabucasin), a small molecule inhibitor that selectively binds to the DBD domain of STAT3, is the only direct STAT3 inhibitor that has advanced into phase III trials thus far. The excellent outcome of a recent phase III monotherapy trial suggested that BBI608 has potential implication in advanced colorectal cancer [117]. Moreover, FDA has approved BBI608 as an orphan drug for treatment of gastric and pancreatic cancer based on the promising results in phase I/II clinical trials.

Numerous small molecule inhibitors of STAT3 have been identified by virtual screening. Of note, although these inhibitors exhibit excellent physicochemical properties *in vitro*, most of them show poor clinical efficacy, which might be due to low aqueous solubility and low cell permeability [86]. Several novel approaches have recently emerged to overcome this dilemma, and show great promise to yield therapeutic agents to targeting transcription factors, including STAT3. For instance, the small-molecule proteolysis-targeting chimera (PROTAC)-based strategy has attracted a lot of attention because it can inhibit target protein function as well as counteract increased target protein expression [128]. The studies on PROTAC-mediated degradation of oncogenic proteins such as BRD4 [129], BCR-ABL [130], receptor tyrosine kinase (RTK) [131], and BCL-X_L_ [132] have shown encouraging results, suggesting the potential clinical applicability of this ingenious approach. SD-36, a novel inhibitor identified by the PROTAC-based strategy, exhibits high selectivity for STAT3 and high cell permeability [98]. Moreover, SD-36 treatment can cause a profound and long-lasting suppression of tumor in mouse models of leukemia and lymphoma [98], suggesting that PROTAC-based strategy may be a promising and reliable avenue for searching small molecule inhibitors against STAT3. Further, the outstanding performance of SD-36 in cancer treatment suggests that the strategy of targeting STAT3 protein degradation may be superior to suppress STAT3 expression.

Oligonucleotides represent a new treatment strategy for ‘undruggable’ cancer targets such as STAT3. STAT3-binding decoy oligodeoxynucleotides, can sequester STAT3 and thus decrease its binding to cognate DNA sites within target genes [133]. Antisense oligonucleotides (ASOs) are designed to block STAT3 activity by targeting STAT3 mRNA. For example, AZD9150, a second-generation STAT3 ASO, targets the 3'-untranslated region (3'-UTR) of the STAT3 gene [134]. Preclinical testing and clinical evaluation have revealed the high efficacy and low toxicity of AZD9150 in oncotherapy [135, 136]. Although oligodeoxynucleotides inhibitors of STAT3 provide exquisite specificity and potency, their poor cell membrane penetrance, rapid degradation, and the lack of effective targeted delivery carriers, remain the major obstacles that impede their use in solid tumors. Aptamers have also emerged as useful targeted delivery agents for conventional drugs and small RNAs including siRNAs and miRNAs due to several advantages, such as small physical size, high stability and low immunogenicity [137]. Recently, STAT3 silencing by aptamer-siRNA chimera obtained excellent inhibition in the therapy of glioblastoma [138, 139], suggesting that the improved oligonucleotides might offer translational potential for the treatment of solid tumors.

Since STAT3 is a transcription factor, it is traditionally regarded as an undruggable target. Direct targeting of STAT3 has proven to be considerably challenging, owing in part to high sequence similarity with the other STAT members [86, 140]. Moreover, several issues such as high toxicity and poor bioavailability have become significant impediments to the clinical development of direct STAT3 inhibitors [86]. Interestingly, some FDA-approved compounds, such as Pyrimethamine and Celecoxib, have been identified as STAT3 inhibitors through drug-repositioning screening [141, 142]. These findings not only provide another source for searching STAT3 inhibitors, but also suggest potential applications of these drugs in cancer therapy. In addition, similar to combined therapy, certain bifunctional compounds are emerging and may represent a new generation of highly efficacious STAT3 inhibitors for cancer therapy in the future. For example, the compound 8u has dual immunotherapeutic and anticancer efficacy through simultaneously inhibiting indoleamine-2,3-dioxygenase 1 (IDO1) and STAT3 [143].

### Indirect targeting of STAT3

In parallel with direct inhibitors, indirect inhibitors of STAT3 have been pursued by targeting the upstream or downstream components of the STAT3 signaling pathway, and hundreds of leading compounds have been identified [144–148]. Out of those, Ruxolitinib, Dasatinib and Siltuximab that target JAK, SRC/ABL, and IL-6 respectively, have been approved by FDA for cancer therapy. Indirect STAT3 inhibitors in currently on-going clinical trials are summarized in Table 2 [120–124]. Of note, indirect STAT3 inhibitors lack specificity for STAT3 and may cause fairly extensive kinase inhibition because the targeted molecules are often involved in intricate signaling pathways.

Intriguingly, it has recently been shown that phosphorylated STAT3 is present in exosomes from 5-fluorouracil (5-FU) resistant colorectal cancer cells, which contributes to acquired 5-FU resistance [149]. Given the importance and efficiency of exosomes in intercellular and interorgan communication [150, 151], these findings not only add another complexity to STAT3 regulation, but also pave a new way to inhibit the oncogenic function of STAT3, as well as to delivery STAT3 inhibitors via exosomes.

## Integrating STAT3 in combination cancer immunotherapy

**Fig. 3:**
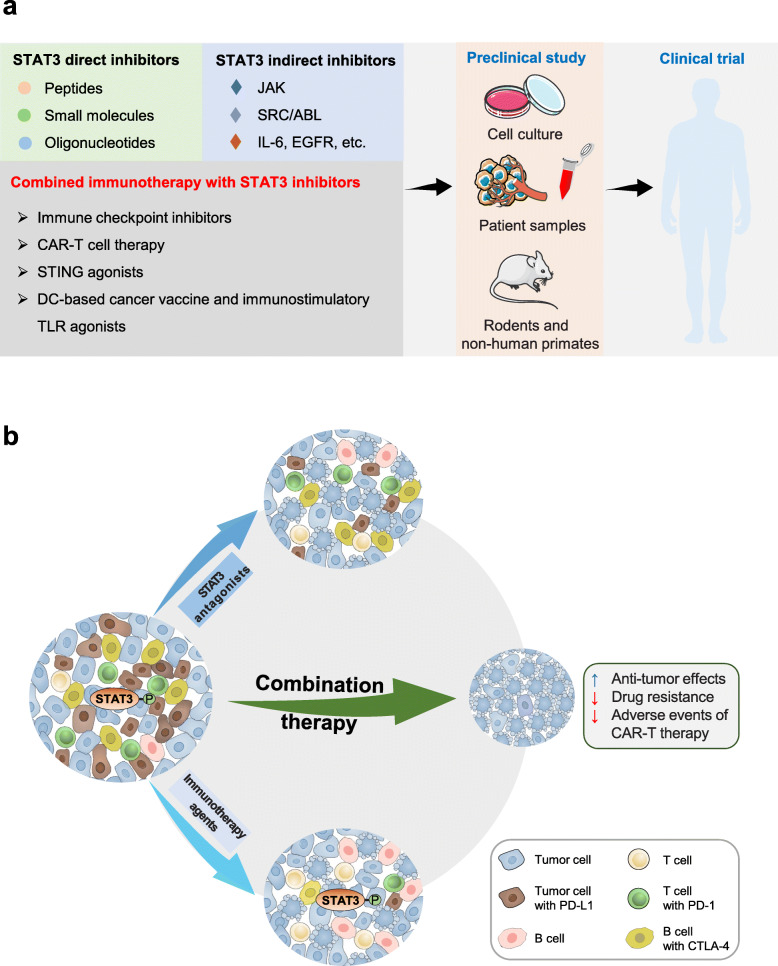
Targeting STAT3 in combination cancer immunotherapy. **a** Summary of the key steps in the development of STAT3-targeting therapeutics. The first step in the development of STAT3-targeting therapeutics involves the systematic selection of STAT3 inhibitors (including direct or indirect inhibitors) and STAT3 inhibitors-based combined immunotherapy, and then elucidating the biology and effects of these candidates to cancer using tumor cell lines and patient samples. The next major challenge involves the *in vivo* model-based validation that these therapeutic candidates must undergo rigorous disease-specific *in vivo* testing using rodents and non-human primate models. Key challenges in translating STAT3 inhibitors into the clinic are low bioavailability and the lack of specific targeting of the tumor site. **b** Targeting STAT3 in combination cancer immunotherapy. Targeting STAT3 in combination cancer immunotherapy can not only enhance the anti-tumor effects, but also reduce drug resistance. Besides, combined STAT3 inhibitors with CAR-T cells can reduce excessive expansion of CAR-T cells and alleviate cytokine release syndrome (CRS), resulting in lower occurrence of immune-related adverse effects.

Immunotherapy is currently among the most promising approaches for cancer treatment. This therapeutic strategy, represented mainly by immune checkpoint blockade (ICB) and chimeric antigen receptor T cells (CAR-T), has obtained unprecedented results in patients with previously incurable cancers [3, 152]. However, there are some key challenges that need to be resolved urgently, including limited clinical response rates and significant autoimmune-related side effects [3, 153]. For instance, ICB has shown remarkable effectiveness in solid tumors including melanoma, non-small cell lung cancers and renal cancer, however, even in these cancers, the majority of patients still do not respond to the treatment [3]. Furthermore, certain types of cancer such as pancreatic cancer and prostate cancer show resistance to immune checkpoint inhibition therapy [3, 154]. Thus, combination therapy is considered to be a promising direction for improving outcomes for cancer treatment. Preclinical and clinical data suggest that combination cancer immunotherapies have enhanced therapeutic efficacy and reduced drug resistance compared with monotherapy [155, 156]. These encouraging data has triggered many investigations of combination strategies, and the combination of STAT3 inhibitors with other immunotherapy agents are also emerging (Fig. 3).

### Combined blockade of STAT3 and immune checkpoint

Up-regulated expression of the immune checkpoint molecules, including CTLA-4, PD-1, and PD-L1, has been documented to facilitate tumor immune escape. A substantial amount of evidence has shown that STAT3 is able to directly or indirectly regulate these immune checkpoint molecules. As a transcription factor, STAT3 can increase expression of PD-1, PD-L1, and PD-L2 by direct binding to their promoters [157–160]. Meanwhile, STAT3 has been identified to indirectly induce expression of immune checkpoint molecules through modulating diverse signaling pathways. For example, STAT3 increased CTLA-4 expression in tumor-associated B cells in a JAK-dependent manner [161] and enhanced CTLA-4 expression in Treg cells through IL-10 [162]. In addition, STAT3 mediated HDAC6-induced PD-L1 expression in osteosarcoma cells [163]. Conversely, recent evidence also suggests a role of immune checkpoint molecules in STAT3 expression. Celada et al. reported that PD-1 upregulation in CD4^+^ T cells leads to an increase in STAT3 mRNA expression by undescribed mechanism, and the latter is required for IL-17 and TGFβ1 production [164]. Interestingly, an early study from the same research group showed PD-1 can attenuate TCR-dependent activation of PI3K/AKT pathway in CD4^+^ T cells [165]. Given PI3K/AKT as a known repressor of STAT3 transcription [166], it is likely that PD-1 indirectly enhances STAT3 expression through inhibition of PI3K. The reciprocal regulation of STAT3 and immune checkpoint molecules not only suggests an involvement of STAT3 in anti-tumor immunity, but also provides a promising strategy to improve the efficacy of current immune checkpoint inhibitors.

Combined blockade of STAT3 and immune checkpoint has shown encouraging results, whereby the addition of STAT3 inhibitors can enhance therapeutic efficacy, and reduce resistance to ICB immunotherapy in parallel. Dasatinib, an indirect STAT3 inhibitor against SRC/ABL, significantly facilitated anti-CTLA-4 immunotherapy in head and neck squamous cell carcinoma [115], while the combined blockade of IL-6 and PD-L1 remarkably inhibited the growth of pancreatic ductal adenocarcinoma and hepatocellular carcinoma (HCC) [167, 168]. The resistance to anti-PD-1 antibodies can be overcome by treatment with JAK inhibitor in mice with pancreatic orthotopic tumors [114]. Niclosamide blocked STAT3-induced PD-L1 transcription, and thus enhanced the efficacy of anti-PD-1/PD-L1 antibodies in non-small cell lung cancer [169]. More recently, a phase III trial reported exciting results of STAT3-based combination therapy in treatment of advanced HCC. Compared to the first-line drug sorafenib, the combination of bevacizumab (a monoclonal antibody targeting VEGF) and atezolizumab (a PD-L1 inhibitor) can significantly prolong the overall survival and progression-free survival of patients with unresectable HCC, along with comparable adverse effects [125]. In addition, certain STAT3 inhibitors, such as BBI608 and AZD9150, combining with immune checkpoint inhibitors, are currently being tested in pre-clinical (Table 1) and clinical trials (Table 2) [126]. The promising early phase clinical trials encourage further clinical development of this combination strategy.

### Combined STAT3 inhibitors and CAR-T

CAR-T cell therapy, a rapidly emerging and effective immunotherapeutic approach, has revolutionized anti-cancer therapies for hematologic malignancies, especially acute lymphoblastic leukemia and lymphoma [152]. Two Anti-CD19 CAR-T therapies are currently approved by FDA for the treatment of CD19-positive leukemia or lymphoma. Although the efficacy of CAR-T therapy in solid tumors has lagged far behind, a great number of CAR-T trials are ongoing and positive outcomes are increasingly being reported for multiple solid tumors, including glioblastoma, gastrointestinal, genitourinary, breast, and lung cancer [170]. For example, CAR-T cells targeting B7-H3, a transmembrane protein belonging to the B7 immune family, inhibited the growth of neuroblastoma, pancreatic and ovarian cancer *in vitro* and in xenograft mouse models without evident toxicity [171, 172].

The involvement of STAT3 signaling in CAR-T therapy is emerging. Transcriptomic profiling showed that anti-CD19 CAR-T cells from responsive patients with chronic lymphocytic leukemia had an increased IL-6/STAT3 signature, which promoted the expansion of CAR-T cells [173]. In line with this, a novel anti-CD19 CAR-T cells with constitutive activation of STAT3 showed increased proliferation and reduced terminal differentiation of CAR-T cells, and conferred superior anti-tumor effects [174]. Similarly, CAR-T cells expressing the ectodomain of the IL-4 receptor and the end domain of the IL-21 receptor activated the STAT3 pathway and enhanced Th17-like polarization, representing a potential clinical CAR-T therapy for solid tumors enriching IL-4 [175]. These studies suggest a beneficial effect of STAT3 activation in CAR-T cells.

As mentioned above, STAT3 hyperactivation in tumor stroma is immunosuppressive and can increase the expression of certain cytokines and growth factors. Accordingly, constitutive expression of an array of cytokines such as IL-6 and IL-10 potentially could increase the risk of serious adverse events of CAR-T therapy including cytokine release syndrome [176]. Thus, there are some attempts to combine CAR-T therapy with STAT3 inhibitors for improving the persistence and anti-tumor effects, as well as negating toxicities of CRA-T cells *in vivo*. For instance, the JAK2/STAT3 axis is a crucial driver of liver-associated MDSCs and inhibition of STAT3 increased the efficacy of CAR-T cells in liver cancer metastasis [177]. In addition, a clinical study is currently on-going, which tests the efficacy and administration of the anti-IL-6 therapy (tocilizumab) on anti-CD19 CAR-T cells associated cytokine release syndrome (NCT02906371).

### Combined STAT3 inhibitors and STING agonists

Stimulator of interferon genes (STING) is a major adaptor protein that plays an important role in anti-viral and anti-tumor immunity [178]. When stimulated by cytosolic DNA, STING activates TANK-binding kinase 1 (TBK1), which subsequently phosphorylates interferon regulatory factor 3 (IRF3) to promote IFN expression [178]. Activated STING can propagate interferon receptor signaling in tumor-infiltrating DCs and elicit CD8^+^ T cells against tumor-associated antigens *in vivo* [179]. Therefore, STING agonists are of continuing research interest as novel adjuvants to boost cancer immunotherapy. A recent study showed that STING-activating nanoparticles (STING-NPs) can convert immunosuppressive tumors to immunogenic microenvironments and then induce anti-tumor immune responses and immunological memory in mice with melanoma [180]. In another study, Ramanjulu et al. reported that STING agonist can lead to complete and lasting regression of tumors in mice with colon tumors [179]. These encouraging results of preclinical studies point towards the potential for improving clinical outcomes of immunotherapy, and some STING agonists such as c-diAM (PS)2 and cGAMP are currently being evaluated in clinical trials (NCT03937141, NCT02986867).

Several previous studies have suggested potential interactions between STING signaling and STAT3-driven oncogenic pathways [181–183]. It has been observed that rapid colorectal cancer progression in STING-deficient mice is associated with STAT3 hyperactivation [181]. Further research found that STING plays a vital role in regulation of MDSC differentiation and anti-tumor immunity in nasopharyngeal carcinoma by increasing the expression of SOCS1, a classic repressor of STAT3 [182]. Besides, TBK1, which is activated by cytosolic DNA in a STING-dependent manner, can restrain activation of STAT3 through direct phosphorylation of STAT3 at serine 754 in the TAD [183].

Recent evidence indicates that the combination of STING agonists with STAT3 inhibitors can enhance tumor immunogenicity and optimize the immunotherapeutic effects [113, 116]. For example, combined STAT3 direct inhibitor HJC0152 with STING agonist c-diAM (PS)2 increased CD8^+^ T cells, reduced Treg cells and MDSCs in the TME, and thus effectively enhanced anti-tumor immunity in mice with breast cancer [113]. A preclinical study demonstrated the combination of STING agonist (cGAMP or RR-CDA) with the indirect STAT3 inhibitor VEGFR2 was maximally effective for immunotherapy-resistant tumors in breast and lung cancer [116].

STAT3 blockade can also markedly improve other effective immunotherapeutic approaches including cancer vaccines and immunostimulatory Toll-like receptor (TLR) agonists (such as CpG oligodeoxynucleotides). For example, a novel strategy combining STAT3 ASO with TLR9 stimulation (CpG oligonucleotide) has been shown to enhance the anti-tumor immunity and overcome tumor immune tolerance in prostate cancer [184]. The combination of STAT3 inhibitor and DC-based vaccine led to improved therapeutic outcomes in mouse colon cancer [185]. The beneficial outcomes of these immunotherapy combinations about STAT3 inhibitors warrant further clinical validation. Notably, STAT3 antagonists, either direct or indirect STAT3 inhibitors, are generally less likely to completely block STAT3 signaling and might not trigger severe autoimmune disorders. However, it cannot be ignored that the use of STAT3 inhibitors and other immunotherapy agents in combination may result in more frequent and severe immune-related adverse events (irAEs) compared to monotherapy. Accordingly, risk evaluations for irAEs should be part of the decision criteria for determining immunotherapy combinations. Likewise, early recognition and adequate management for irAEs are indispensable to minimize treatment-related serious complications.

## Conclusions and perspectives

STAT3 becomes excessively activated in multiple human cancers, and acts as a crucial signaling node for tumor cells and TME comprising cells, especially tumor-infiltrating immune cells. Therefore, targeting STAT3 is expected to offer multiple benefits, including reduced tumor cell intrinsic proliferation, enhanced anti-tumor effects of tumor-infiltrating immune cells, and improved the immunosuppressive crosstalk within the TME. These effects have positioned STAT3 as an arisen potential promising target for cancer therapy.

To date, many endeavors have been made to target STAT3 for the development and application of new drugs. These approaches are devised to inhibit STAT3 directly by peptides, small molecules and decoy oligonucleotides, or indirectly by blocking upstream signaling pathways such as IL-6 and JAK2 pathways. Currently, the core idea of direct targeting STAT3 is to prevent the formation of functional STAT3 dimers through disrupting phosphorylation of STAT3. Beyond phosphorylation, other posttranslational modifications, such as acetylation, methylation and sumoylation, are emerging to modulate STAT3 activation through diverse mechanisms, providing a broadened list of candidate regulatory targets for the STAT3 inhibitors.

To improve the response rate and the number of responding cancer types, combined immunotherapies are now being undertaken. Combination therapies of STAT3 inhibitors with currently therapeutic anti-tumor drugs including the immune-checkpoint inhibitors may open up new possibilities for long-lasting and multilayered tumor control. Although preclinical studies and early clinical trials on combined blockade of STAT3 and immune checkpoint have shown encouraging results, their clinical outcomes await further investigation. Moreover, predictive biomarkers are urgently required to rationally incorporate STAT3 inhibitors into the combination immunotherapy. The ncRNAs, particularly miRNAs, might prove to be potentially promising predictive biomarkers that can provide a basis for improved precision medicine, though the related studies are currently not explored in depth.

In summary, therapeutically targeting mediators of the STAT3 signaling, which has already been shown to be beneficial in the restoration of anti-tumor immunity, provides attractive avenues that are currently being explored for the immunotherapy of cancers both as monotherapy and in combination therapies.

## Data Availability

Not applicable.

## Abbreviations

AHR: Aryl hydrocarbon receptor
ALL: Acute lymphoblastic leukemia
AOM: Azoxymethane
ASOs: Antisense oligonucleotides
BC: Breast cancer
Bcl-2: B-cell lymphoma-2
BRD4: Bromodomain-containing protein 4
CAFs: Cancer-associated fibroblasts
CAR-T: Chimeric antigen receptor T cells
CCD: Coiled-coil domain
CCL: C-C motif ligand
CD80/86: Cluster of differentiation 80/86
circRNAs: Circular RNAs
CLL: Chronic lymphocytic leukemia
CML: Chronic myelogenous leukemia
CRS: Cytokine release syndrome
CTLA-4: Cytotoxic T-lymphocyte-associated protein 4
CXCL: C-X-C motif chemokine ligand
Cxxc1: Cxxc finger protein 1
DBD: DNA-binding domain
DCs: Dendritic cells
DSS: Dextran sodium sulfate
DUSP22: Dual specificity phosphatase 22
EGF: Epidermal growth factor
EGFR: Epidermal growth factor receptor
EZH2: Enhancer of zeste homolog 2
FGFR: Fibroblast growth factor receptor
GAC: Gastric adenocarcinoma
GC: Gastric cancer
GIST: Gastrointestinal stromal tumor
GP130: Glycoprotein 130
HADC: Histone deacetylase
HCC: Hepatocellular carcinoma
HIF1α: Hypoxia-inducible factor 1α
HNSCC: Head and neck squamous cell carcinoma
hpdODN: Hairpin decoy oligodeoxynucleotide
ICB: Immune checkpoint blockade
IDO1: Indoleamine-2,3-dioxygenase 1
IFNs: Interferons
IFN-Is: Type I interferons
IL: Interleukin
Loxl3: Lysyl oxidase-like 3
irAEs: Immune-related adverse events
IRF3: Interferon regulatory factor 3
ISGF3: Interferon-stimulated gene factor 3
JAKs: Janus kinases
LLC: Lewis lung carcinoma
lncRNAs: Long non-coding RNAs
LIF: Leukemia inhibitory factor
MDSCs: Myeloid-derived suppressor cells
MHC class II: Major histocompatibility complex class II
MMPs: Matrix metalloproteinases
MPLW515L: Somatic mutations at codon 515 of the thrombopoietin receptor
ncRNAs: Non-coding RNAs
NH2: Amino-terminal domain
NSCLC: Non-small cell lung cancer
NF-κB: Nuclear factor-κB
NTD: N-terminal domain
PC: Pancreatic cancer
PCa: Prostate cancer
PD-1: Programmed cell death protein 1
PDGFR: Platelet-derived growth factor receptor
PD-L1: Programmed cell death 1 ligand 1
PKCβII: Protein kinase C β II
PMF: Primary myelofibrosis
PIAS: Protein inhibitor of activated STAT
PlGF: Placenta growth factor
PI3K: Phosphatidylinositol 3 kinase
Post-ET MF: Post-essential thrombocythemia myelofibrosis
Post-PV: Post-polycythemia vera
PROTAC: Proteolysis-targeting chimera
PTPases: Protein tyrosine phosphatases
PTPN1: Protein tyrosine phosphatase non-receptor type 1
PTPRD: Protein tyrosine phosphatase receptor type D
PTPRT: Protein tyrosine phosphatase receptor type T
RTK: Receptor tyrosine kinase
SH2: Src homology 2 domain
SHP: Src homology 2 domain containing protein tyrosine phosphatase
SIRT1: Sirtuin 1
SMYD2: SET (Suppressor of variegation, Enhancer of Zeste, Trithorax) and MYND (Myeloid-Nervy-DEAF1) domain containing 2
SOCS: Suppressor of cytokine signaling
STAT: Signal transducer and activator of transcription
STING: Stimulator of interferon genes
STING-NPs: STING-activating nanoparticles
SUMO2/3: Small ubiquitin-like modifier 2/3
TAD: Transactivation domain
TBK-1: TANK-binding kinase 1
TCR: T cell receptor
TGFβ: Transforming growth factor β
Th1: T helper 1
TLR: Toll-like receptor
TME: Tumor microenvironment
TNFα: Tumor necrosis factor α
Treg: Regulatory T-cell
VEGF: Vascular endothelial growth factor
VEGFR: Vascular endothelial growth factor receptor
3ʹ-UTR: 3ʹ-untranslated region
5-FU: 5-fluorouracil
